# BoHV-4-based vector delivering Ebola virus surface glycoprotein

**DOI:** 10.1186/s12967-016-1084-5

**Published:** 2016-11-24

**Authors:** Alfonso Rosamilia, Sarah Jacca, Giulia Tebaldi, Silvia Tiberti, Valentina Franceschi, Francesca Macchi, Sandro Cavirani, Gary Kobinger, Donald P. Knowles, Gaetano Donofrio

**Affiliations:** 1 Department of Medical-Veterinary Science, University of Parma, Via del Taglio 10, 43126 Parma, Italy; 2 Special Pathogens Program, University of Manitoba and Public Health Agency of Canada, Winnipeg, MB Canada; 3 Animal Disease Research Unit, Agricultural Research Service, United States Department of Agriculture, and Department of Veterinary Microbiology & Pathology, Washington State University, Pullman, WA USA

**Keywords:** Ebola virus, Bovine herpesvirus 4, Vaccine platform, Viral vector, Recombineering

## Abstract

**Background:**

Ebola virus (EBOV) is a Category A pathogen that is a member of *Filoviridae* family that causes hemorrhagic fever in humans and non-human primates. Unpredictable and devastating outbreaks of disease have recently occurred in Africa and current immunoprophylaxis and therapies are limited. The main limitation of working with pathogens like EBOV is the need for costly containment. To potentiate further and wider opportunity for EBOV prophylactics and therapies development, innovative approaches are necessary.

**Methods:**

In the present study, an antigen delivery platform based on a recombinant bovine herpesvirus 4 (BoHV-4), delivering a synthetic EBOV glycoprotein (GP) gene sequence, BoHV-4-syEBOVgD106ΔTK, was generated.

**Results:**

EBOV GP was abundantly expressed by BoHV-4-syEBOVgD106ΔTK transduced cells without decreasing viral replication. BoHV-4-syEBOVgD106ΔTK immunized goats produced high titers of anti-EBOV GP antibodies and conferred a long lasting (up to 6 months), detectable antibody response. Furthermore, no evidence of BoHV-4-syEBOVgD106ΔTK viremia and secondary localization was detected in any of the immunized animals.

**Conclusions:**

The BoHV-4-based vector approach described here, represents: an alternative antigen delivery system for vaccination and a proof of principle study for anti-EBOV antibodies generation in goats for potential immunotherapy applications.

**Electronic supplementary material:**

The online version of this article (doi:10.1186/s12967-016-1084-5) contains supplementary material, which is available to authorized users.

## Background

Ebola virus (EBOV) is a pathogen responsible of outbreaks of human hemorrhagic fever in African countries, including the last epidemic, which ended with more than 11,300 deaths in Guinea, Liberia and Sierra Leone (updated since September 20th; http://apps.who.int/ebola/ebola-situation-reports). EBOV infection is characterized by systemic viral replication, host immunity hyper responsiveness along with a cytokine storm and disseminated intravascular coagulation similar to septic shock [1]. EBOV belongs to the *Filoviridae* family which includes two genera, Ebolavirus and Marburgvirus. The genus *Ebolavirus* includes three species pathogenic in humans, *Zaire ebolavirus* [case fatality report (CFR) 70–90%]; *Sudan ebolavirus* (CFR ~50%) and *Bundibugyo ebolavirus* (CFR ~25%) [2].

Vaccine production and availability is widely dependent on commercial factors. Indeed, it is not a mere coincidence if vaccines dedicated to important diseases of undeveloped countries are less prevalent on the market than those for diseases of developed countries. An important exception could be represented by EBOV vaccines. Although the disease has been known by the scientific community since 1976, an effective, commercially available vaccine is still lacking. The recent EBOLA outbreak, which began in December 2013, affected both people in isolated rural areas and in large cities. The outbreak reached global dimensions and EBOV-infected patients have been hospitalized not only in Africa but also in USA and Europe. This phenomenon captured the attention of the global scientific community. However, research activity in this field is hampered by the need of costly facilities which is the most important issue in dealing with infectious pathogens for which there are few available vaccines and no effective treatment.

So far a dozen vaccines proved effective protection in non-human primates from lethal EBOV infection and several ones are in advanced trial phases. Most of these vaccine approaches are viral vector-based, where the immune-dominant full length membrane glycoprotein (GP) open reading frame is delivered by a recombinant viral vector. Platforms based on recombinant adenovirus serotype 5 (rAd5) vectors [3], combined DNA/rAd5 vectors [4], combined rAd serotype 26 and 35 vectors, recombinant chimpanzee adenovirus serotype 3 (rChAd3) vectors [3], alphavirus replicons based on recombinant human parainfluenza virus 3 (rHPIV3) [5], rabies virus [6], and recombinant vesicular stomatitis virus (sVSV) [7], have been exploited with successful results [8]. Vectorialized viruses are not only mere delivery systems but also a sort of adjuvant which strongly induce an active immunity. There are several types of viral vectors, derived from different classes of viruses and each of them possess particular characteristics. It is therefore difficult to predict which virus will best achieve the vaccine-vector goal. It must be kept in mind that a specific viral-vector could be suitable for the immunization toward a specific pathogen, but not toward others. Consequently, it would be of great interest to explore new vaccine-vector agents based on different viruses. Bovine herpesvirus 4 (BoHV-4)-is a relatively new viral vector derived from bovine *gammaherpesvirus*. Recombinant BoHV-4s cloned as bacterial artificial chromosome (BAC), delivering ORFs coding for immune-dominant antigens from different pathogens, were shown to successfully elicit a functional immune-response in mice [9, 10], rats [11], rabbits [12], sheep [13], swine [14] and goats [15]. In the present paper, a BoHV-4-based vector platform was generated exploiting a synthetic gene approach; a recombinant BoHV-4 delivering EBOV GP ORF expression cassette was constructed and goats were successfully immunized.

## Methods

### Cell lines

Bovine embryo kidney [(BEK) were from Dr. M. Ferrari, Istituto Zooprofilattico Sperimentale, Brescia, Italy; (BS CL-94)], BEK expressing cre recombinase (BEK cre) [16] and human embryo kidney 293T [(HEK 293T) ATCC: CRL-11268] cell lines were cultured in complete growth medium Eagle’s minimal essential medium (EMEM, LONZA) containing 10% fetal bovine serum (FBS), 2 mM of l-glutamine (SIGMA), 100 IU/mL of penicillin (SIGMA), 100 μg/mL of streptomycin (SIGMA) and 2.5 μg/mL of Amphotericin B (SIGMA) and incubated at 37 °C, 5% CO_2_.

### Constructs

Synthetic Zaire Ebola virus Mayinga glycoprotein (GP) ORF, tagged at the carboxy-terminal with gD106 peptide (syEBOVgD106) was excised from pUC57sy EBOVgD106 (EUROFINS, GENOMICS) with *Nhe*I and *Sma*I enzymes and the 2153 bp fragment was inserted inside *Nhe*I/*Sma*I cut pINT2EGFPTK shuttle vector [17] to generate pINT2CMVsyEBOVgD106. EBOV secreted fragment (EBOVsec), without the trans-membrane domain, was obtained by amplification from pINT2CMVsyEBOVgD106 with *Nhe*I EBOGP sense (5′- ggggctagcccaccatgggcgtg-3′) and *Sal*I EBOGP antisense (5′-ggggtcgacctggcgccagccggtccaccagtt 3′) primers. The generated 1967 bp *Nhe*I-EBOsec-*Sal*I was inserted inside *Nhe*I/*Sal*I digested pIgkE2gD106 to obtain the pCMVEBOsecgD106 construct.

### Transient transfection

Confluent HEK 293T cells were seeded into six well plates (3 × 10^5^ cells/well) and incubated at 37 °C with 5% CO_2_. When the cells were sub-confluent, the culture medium was removed and the cells were transfected with pINT2CMVsyEBOVgD106 using polyethylenimine (Pei) transfection reagent (POLYSCIENCES, INC.). Briefly, 3 μg of DNA were mixed with 7.5 μg PEI (1 mg/mL) (ratio 1:2.5 DNA-Pei) in 200 μL of Dulbecco’s modified essential medium (DMEM) high glucose (EUROCLONE) without serum. After 15 min at RT, 800 μL of medium without serum were added and the transfection solution was transferred to the cells and left for 6 h at 37 °C with 5% CO_2_, in a humidified incubator. The transfection mixture was then replaced with fresh medium (EMEM, with 10% FBS, 50 IU/mL of penicillin, 50 μg/mL of streptomycin and 2.5 μg/mL of Amphotericin B) and incubated for 24 h at 37 °C with 5% CO_2_.

### Viruses and viral replication

BoHV-4-syEBOVgD106ΔTK and BoHV-4-A were propagated by infecting confluent monolayers of BEK cells at a multiplicity of infection (MOI) of 0.5 tissue culture infectious doses 50 (TCID50) per cell and maintained in medium with only 2% FBS for 2 h. The medium was then removed and replaced with fresh EMEM containing 10% FBS. When the cytopathic effect (CPE) affected the majority of the cell monolayer (~72 h post infection), the virus was prepared by freezing and thawing cells three times and pelleting the virions through a 30% sucrose cushion, as described previously [18]. Virus pellets were then resuspended in cold EMEM without FBS. TCID_50_ were determined with BEK cells by limiting dilution.

### Semi-reducing western immunoblotting

Protein cell extracts were obtained from a six-well confluent plate of HEK 293T transfected with pINT2CMVsyEBOVgD106 and from 25-cm^2^ confluent flasks of BEK infected with BoHV-4- syEBOVgD106ΔTK by adding 100 μL of cell extraction buffer (50 mM Tris–HCl, 150 mM NaCl, and 1% NP-40; pH 8). A 10% SDS-PAGE gel electrophoresis was used to analyze cell extracts containing 50 μg of total protein, after protein transfer in nylon membranes by electroblotting, the membranes were incubated with primary bovine anti BoHV-1 glycoprotein D monoclonal antibody (clone 1B8-F11; VRMD, Inc., Pullman, WA, USA), diluted 1:15.000, and then with a secondary antibody probed with horseradish peroxidase-labelled anti-mouse immunoglobulin (SIGMA), diluted 1:10.000, to be visualized by enhanced chemiluminescence (ECL KIT; PIERCE). Cell supernatant obtained from HEK 293T transfected with pCMVEBOsecgD106 was collected at different time points (16, 24, 40, 48, 50, 60 and 70 h after transfection) and analyzed as above.

### BAC recombineering and selection

Recombineering was performed as previously described [19] with some modifications. After recombineering, only those colonies that were kanamycin negative and chloramphenicol positive were kept and grown overnight in 5 mL of LB containing 12.5 mg/mL of chloramphenicol. BAC DNA was purified and analyzed through *Hin*dIII restriction enzyme digestion. DNA was separated by electrophoresis in a 1% agarose gel, stained with ethidium bromide, and visualized through UV light. Original detailed protocols for recombineering can also be found at the recombineering website (http://recombineering.ncifcrf.gov).

### Southern blotting

DNA from 1% agarose gel was capillary transferred to a positively charged nylon membrane (ROCHE), and cross-linked by UV irradiation by standard procedures [16].

The membrane was pre-hybridized in 50 mL of hybridization solution (7% SDS, 0.5 M phosphate, pH 7.2) for 1 h at 65 °C in a rotating hybridization oven (TECHNA INSTRUMENTS). The 1967 bp amplicon for EBO digoxigenin-labeled probe was generated by PCR with *Nhe*I EBOGP sense (5′-ggggctagcccaccatgggcgtg-3′) and *Sal*I-EBOGP antisense (5′-ggggtcgacctggcgccagccggtccaccagtt 3′) primers, as previously described [12].

### Cell culture electroporation and recombinant virus reconstitution

BEK or BEK cre cells were maintained as a monolayer with complete DMEM growth medium with 10% FBS, 2 mM l-glutamine, 100 IU/mL penicillin and 10 mg/mL streptomycin. When cells were sub-confluent (70–90%) they were split to a fresh culture flask (i.e., every 3–5 days) and were incubated at 37 °C in a humidified atmosphere of 95% air–5% CO_2_. BAC DNA (5 μg) was electroporated in 600 μL DMEM without serum (EQUIBIO APPARATUS, 270 V, 960 mF, 4-mm gap cuvettes) into BEK and BEK cre cells from a confluent 25-cm^2^ flask. Electroporated cells were returned to the flask, after 24 h the medium was replaced with fresh medium, and cells were split 1:2 when they reached confluence at 2 days post-electroporation. Cells were left to grow until the appearance of CPE. Recombinant viruses were propagated by infecting confluent monolayers of BEK cells at a M.O.I. of 0.5 TCID50/cell and maintaining them in EMEM with 10% FBS for 2 h.

### Viral growth curves

BEK cells were infected with BoHV-4-A and BoHV-4syEBOVgD106ΔTK at a M.O.I. of 0.1 TCID50/cell and incubated at 37 °C for 4 h. Infected cells were washed with serum-free EMEM and then overlaid with EMEM containing 10% FBS, 2 mM l-glutamine, 100 IU/mL penicillin, 100 mg/mL streptomycin and 2.5-mg/mL Amphotericin B. The supernatants of infected cultures were harvested after 24, 48, 72 and 96 h, and the amount of infectious virus was determined by limiting dilution on BEK cells.

### Samples collection and ELISA procedure

Blood samples were processed for ELISA test. Briefly, microplates (MICROLON HIGH BINDING) were coated overnight at 4 °C with 50 ng/well EBOsecgD106 protein supernatant obtained from 175-cm^2^ sub-confluent HEK 293T cells, transfected with pCMVEBOsecgD106 (Additional file 1: Figure S1) and diluted in 0.1 M carbonate/bicarbonate buffer pH 9.6. After blocking with 1% bovine serum albumin (BSA), serum samples at different dilutions (1/10, 1/100, 1/1000 and 1/10,000) were incubated for 1 h at room temperature. After three washing steps, 50 μL of donkey anti-goat IgG-HRP (SANTA CRUZ BIOTECNOLOGY, Germany) diluted 1:1.000 was added to each well and the plate was incubated as above. Following the final washing step, the reaction was developed with 3,3′,5,5′-tetramethylbenzidine (TMB), stopped with 0.2 M H_2_SO_4_ and read at 450 nm.

## Results

### In silico design of an EBOLA virus tagged ORF

**Fig. 1:**
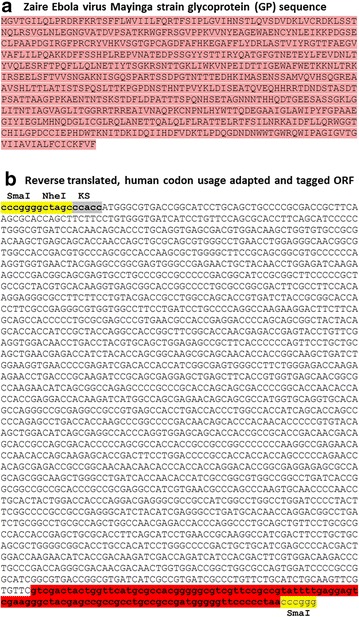
In silico design of EBOV GP synthetic ORF. **a** Zaire EBOV Mayinga strain GP sequence and **b** deduced nucleotide sequence provided of a tag peptide (gD 106; highlighted in *red*) a Kozak’ s sequence (KS; highlighted in *grey*) and restriction enzyme sites (*Sma*I and *Nhe*I; highlighted in *yellow*) for subcloning

The Zaire Ebola virus Mayinga strain glycoprotein (GP) sequence, identical to the Gabon-94 strain and highly conserved with the new isolates (accession number: KJ660346.2, KJ660347, KJ660348), was used as a template to obtain its nucleotide sequence by reverse translation, which was human codon usage adapted and tagged at the carboxy-terminal with gD106 tag peptide [10, 20] (Fig. 1a, b). Furthermore, a Kozak’s sequence (to improve translation) and restriction enzyme sites (to facilitate the sub-cloning in suitable vector) were added to the synthetic ORF antigen (Fig. 1b), which we termed “syEBOVgD106”.

### Generation and expression of syEBOVgD106 antigen expression cassette

**Fig. 2:**
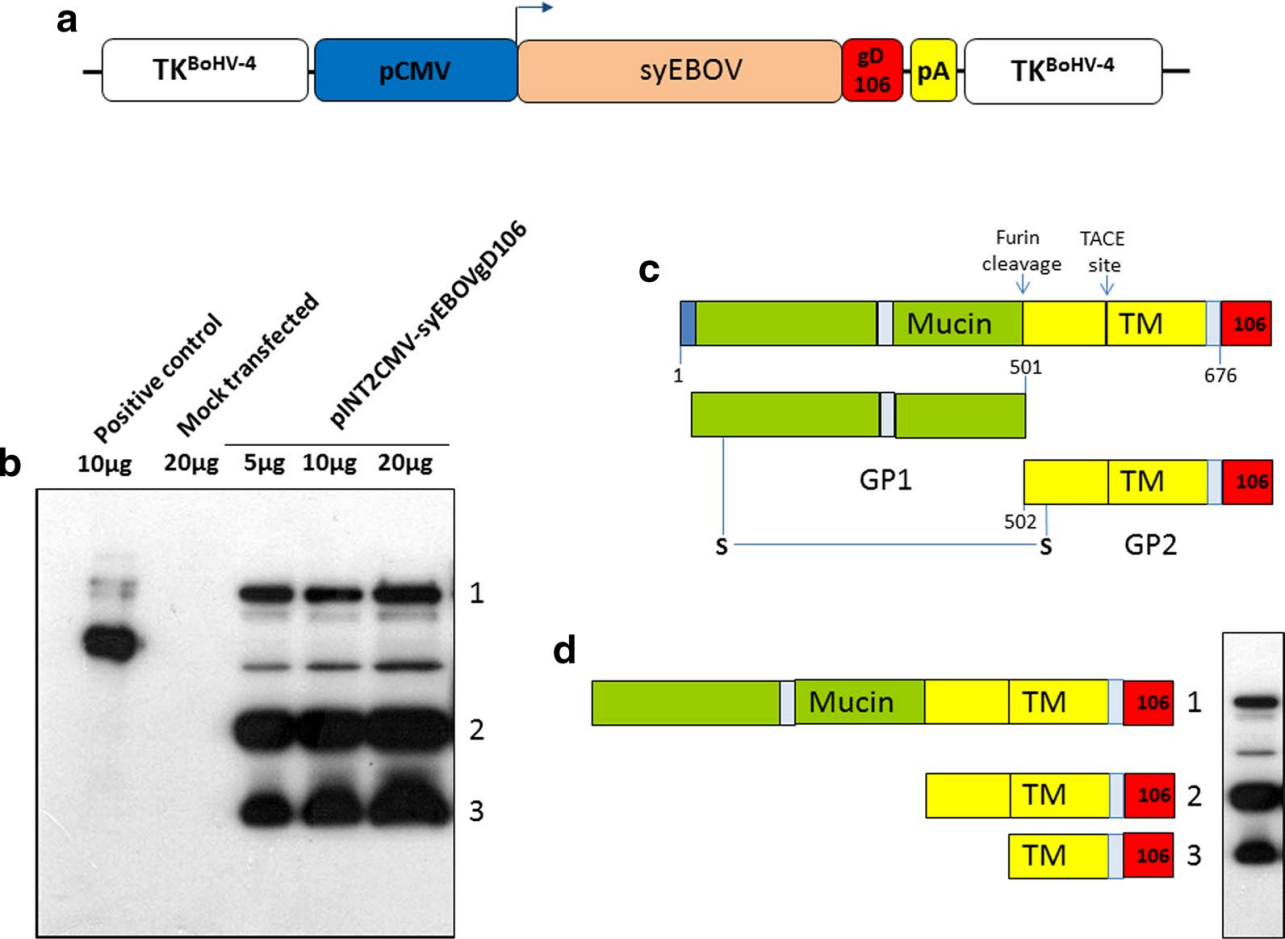
Expression of syEBOVgD106. **a** Diagram (not on scale) of pINT2CMV-syEBOVgD106 targeting vector delivering the tagged syEBOVgD106 ORF (syEBOV, *orange*; gD106, *red*), under the control of the CMV promoter (CMV, *blue*) and the bovine growth hormone polyadenylation signal (PA, *yellow*). CMV-syEBOVgD106 expression cassette is flanked by BoHV-4 TK homologous sequences (*white*). **b** Western immunoblotting of pINT2CMV-syEBOVgD106 transfected HEK 293T cells extracts. *Lanes* were loaded with different amounts of total protein cell extract (5, 10 and 20 μg); cells transfected with pEGFPC-1 served as negative controls (*Mock*). The peculiar immune-blotting banding pattern is the result of the syEBOVgD106 protein processing by furin and TACE proteases (**c**), as revealed by the anti gD106 tag monoclonal antibody directed against three predicted and detected peptides (*1* uncleaved; *2* only cleaved by Furin protease; *3* cleaved by Furin and TACE proteases) (**d**)

syEBOVgD106 synthetic ORF was cut out from its plasmid back-bone and sub-cloned in pINT2EGFP [17] shuttle vector containing a CMV-EGFP expression cassette, where EGFP ORF was substituted with syEBOVgD106 ORF. The resulting construct, pINT2CMV-syEBOVgD106, has two BoHV-4 TK region sequences, flanking the CMV-syEBOVgD106 expression cassette under the transcriptional control of the CMV early promoter and the bovine growth hormone polyadenylation signal sequence (Fig. 2a). The pINT2CMV-syEBOVgD106 construct was validated in terms of protein expression by transient transfection assay in HEK 293T cells and by western blotting using a specific monoclonal antibody against gD106 tag peptide. As expected, syEBOVgD106 glycoprotein was very well expressed in the pINT2CMV-syEBOVgD106 transfected HEK 293T cells (Fig. 2b) and showed the predicted banding pattern (Fig. 2c, d) generated by the cellular metalloprotease TACE [TNFα-converting enzyme, a member of the ADAM (a disintegrin and metalloproteinase proteinase family)] and Furin [21, 22].

### Vectorization of syEBOVgD106 expression cassette in BoHV-4-based vector

**Fig. 3:**
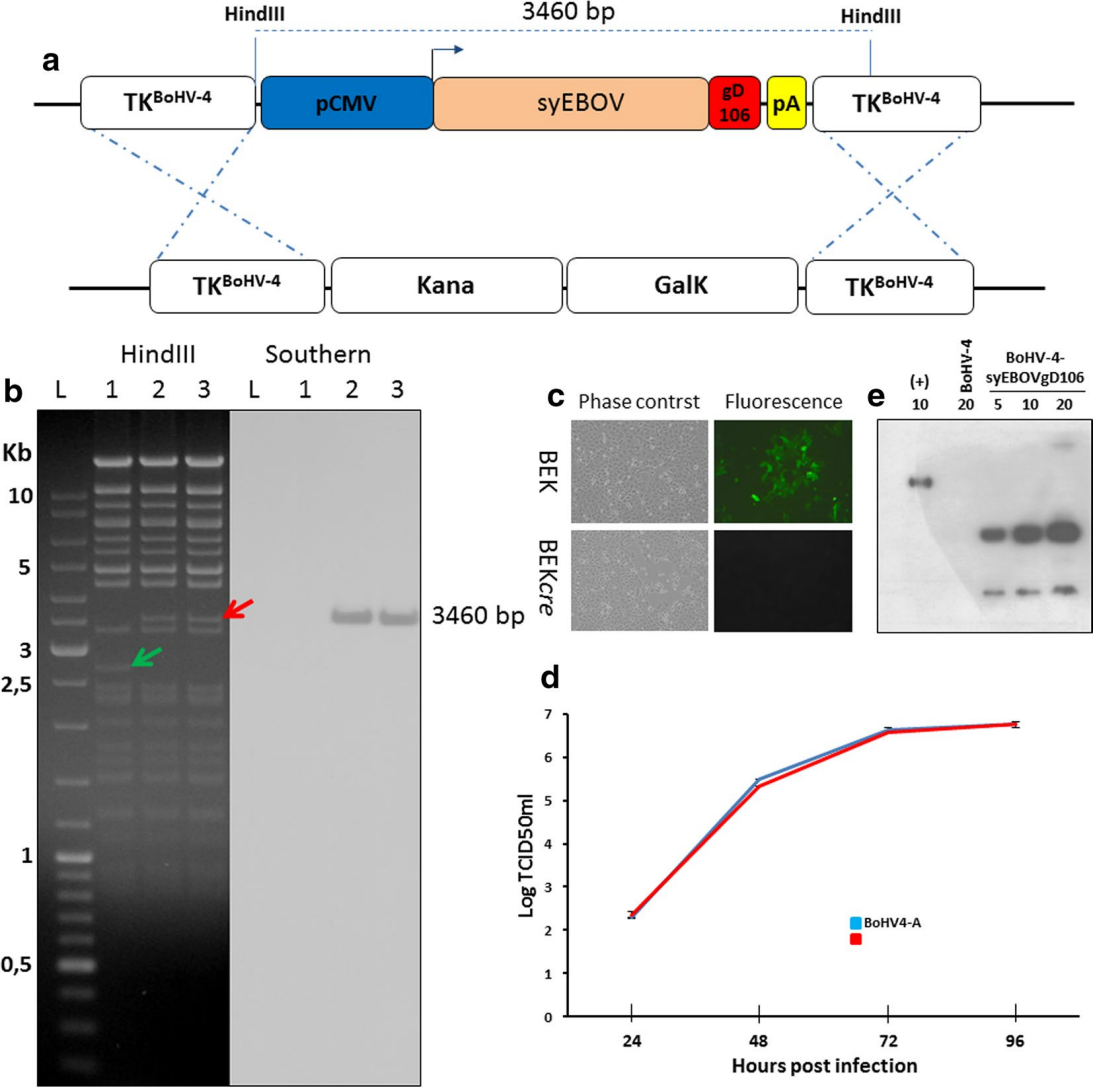
BoHV-4-syEBOVgD106ΔTK constructs and characterization. **a** Diagram (not to scale) illustrating the re-targeting event (i.e., replacement of the Kana/GalK cassette with the CMV-syEBOVgD106 cassette) obtained by heat-inducible homologous recombination in SW102 *E. coli* cells containing pBAC-BoHV-4-A-TK-KanaGalK-TK. **b** Representative, 2-deoxy-galactose resistant colonies, tested by *Hin*dIII restriction enzyme analysis and southern blotting performed with a specific probe for syEBOVgD106 ORF. The 2650 bp band (indicated by a *green arrow*) corresponding to the non-retargeted pBAC-BoHV-4-A-TK-KanaGalK-TK control (*lane 1*) is replaced by 3460 bp band (indicated by a *red arrow*) in pBAC-BoHV-4-syEBOVgD106ΔTK (*lanes 2* and *3*). Phase contrast and fluorescent microscopy images of the plaques formed by viable, reconstituted recombinant BoHV-4-syEBOVgD106ΔTK (**c**) after electroporation of the corresponding BAC DNA clones into BEK or *BEKcre* cells (magnification, ×10). **d** Replication rate of BoHV-4-syEBOVgD106ΔTK grown in BEK cells and compared with that of the parental BoHV-4-A isolate. The data are the mean ± standard error of triplicate measurements (*P* > 0.05 for all time-points; Student’s *t* test). **e** Immunoblotting analyses conducted on extracts from cells infected with BoHV-4-syEBOVgD106ΔTK (*numbers* indicate the micrograms of total protein loaded). BoHV-4-A infected cells served as negative controls

pINT2CMV-syEBOVgD106 shuttle plasmid construct was employed to generate pBAC-BoHV-4-syEBOVgD106ΔTK by heat-inducible homologous recombination in SW102 *E. coli* containing pBAC-BoHV-4-A-KanaGalKΔTK (Fig. 3a). KanaGalK negative selected colonies were amplified in liquid media and their respective BAC analyzed by *Hin*dIII restriction enzyme and successively by southern hybridization (Fig. 3b) using a specific, non-isotopic labeled probe directed against syEBOVgD106 ORF. pBAC-BoHV-4-syEBOVgD106ΔTK stability was assessed by host bacterial serial passages (over 20) and absence of aberrant recombination was detected by restriction enzyme digestion (data not shown).

Infectious BoHV-4-syEBOVgD106ΔTK viral particles were obtained by transfecting, through electroporation, BEK cells or BEK cells expressing *cre* recombinase (BEK*cre*) [16] to deplete the BAC cassette from pBAC-BoHV-4-syEBOVgD106ΔTK. In both cases, viable BoHV-4-syEBOVgD106 producing CPE and plaques was generated, although BoHV-4-syEBOVgD106ΔTK produced in BEK*cre* lost GFP expression due to the removal of GFP expression cassette associated to the BAC plasmid back-bone (Fig. 3c). Next, the growth characteristics of BoHV-4-syEBOVgD106ΔTK were compared with that one of the BoHV-4-A parental virus and no differences between them were observed (Fig. 3d). Furthermore, BoHV-4-syEBOVgD106ΔTK infected cells expressed syEBOVgD106 glycoprotein (Fig. 3e). Since the EBOV GP is a typical, type 1 integral membrane glycoprotein, its ability to be incorporated on the BoHV-4 envelope was investigated. A virus preparation from BoHV-4-A-CMV-IgK-gE2gD-TM was purified and analyzed by Western immunoblotting (Additional file 2: Figure S2). As expected, EBOV GP signal was detected only in BoHV-4-syEBOVgD106ΔTK virions, but not in the wild-type BoHV-4 virions, thus indicating the incorporation of syEBOVgD106 into BoHV-4 virus particles.

### Goat immunization and humoral immune-response analysis

**Fig. 4:**
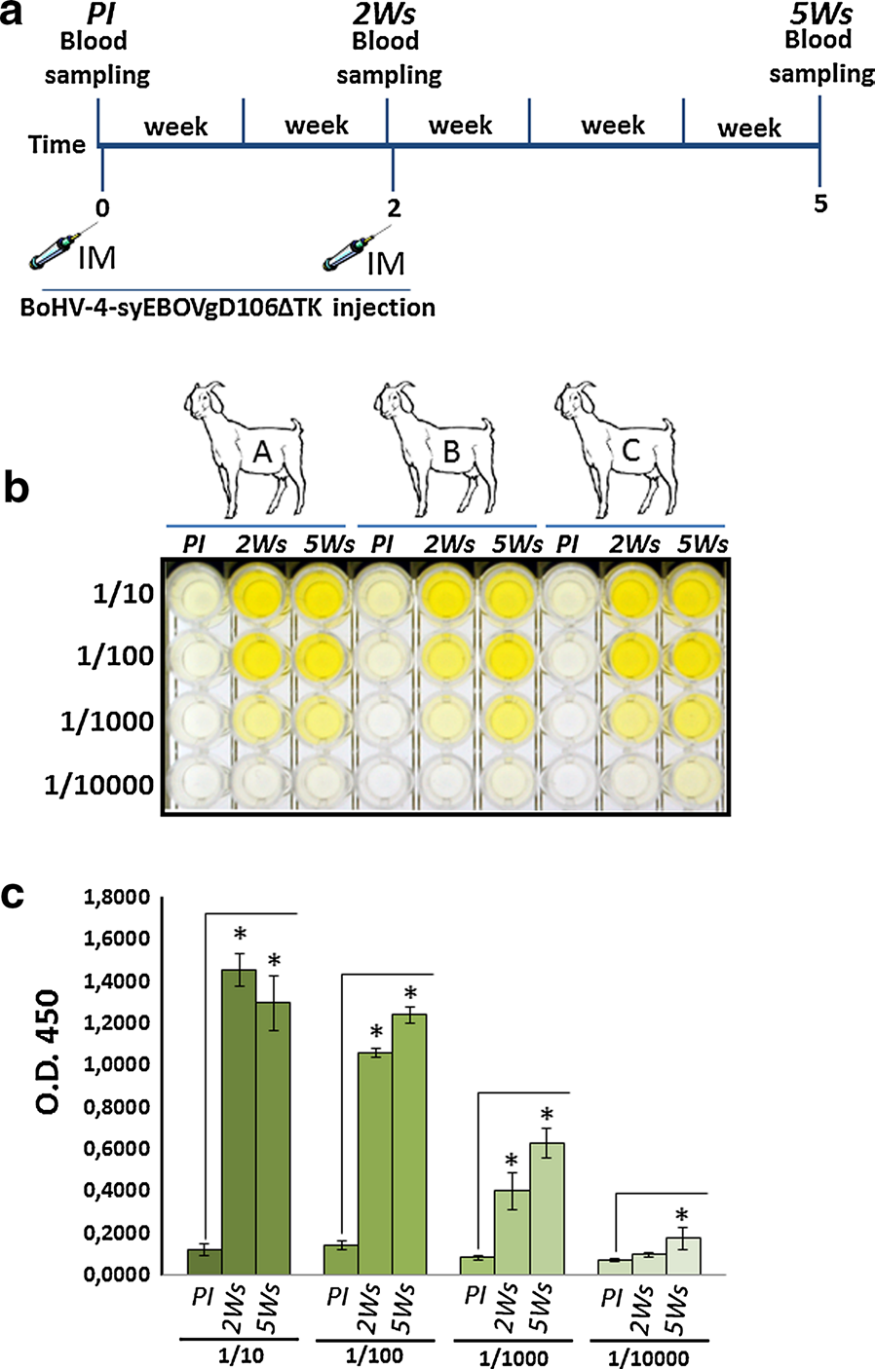
Kinetics of the humoral immune responses of goats immunized with BoHV-4-syEBOVgD106ΔTK. **a** Diagram showing the goats immunization scheme and blood sample collection. **b** Dilutes sera (1/10, 1/100, 1/1000 and 1/10,000) collected at 2 and 5 weeks (2 Ws; 5 Ws) from three (*A*, *B* and *C*) BoHV-4-syEBOVgD106ΔTK inoculated goats, were compared with pre-immune sera (PI) for anti-GP antibodies by ELISA. **c** Antibodies detected were expressed as the optical density at 450 nm and each value represents the mean response of the three goats sera, ± the standard error of the mean, at the same dilution and collected at the same time. Response differences between pre- and post-immune sera were measured by Student’s *t* test (*P ≤ 0.005)

For testing the ability of BoHV-4-syEBOVgD106ΔTK to induce a humoral immune response in a large animal model, a pilot immunization study was performed in goats. In agreement with the current legislation on animal experimentation, which suggests to minimize the number of animals employed, three adult goats, after collection of the pre-immune serum, were inoculated subcutaneously with 1 mL of 10^6^ TCID50 of BoHV-4-syEBOVgD106ΔTK. A second inoculation with an identical dose of BoHV-4-syEBOVgD106ΔTK was done 2 weeks apart from the first inoculation. Blood samples were collected at two, just before the second inoculum, and 5 weeks from the first inoculum (Fig. 4a). Serum samples were analyzed by ELISA and all three goats developed a very good antibody response, with high titers (Fig. 4b, c). As previously described for BoHV-4-based vaccine vector inoculation in goats [15], in the present study, a single inoculation of BoHV-4-syEBOVgD106ΔTK was able to elicit a humoral immune response 2 weeks post inoculation. Moreover, the antibody titer did not decrease when evaluated 6 months later (data not shown), showing long lasting immunization.

## Discussion

In this work, the potential utility of BoHV-4 as a safe, potent, large-capacity gene delivery vector for EBOV antigen was shown. A workflow strategy to construct a BoHV-4-based vector was generated and it was able to deliver an immune-dominant antigen derived from a BSL4 pathogen, thus avoiding all the economical and safety requirements necessary for the manipulation of this kind of biological agent. Furthermore, it was able to elicit a strong humoral immune-response. The results were obtained through a synthetic gene approach, currently based on solid-phase DNA synthesis, which allows the complete synthesis of a double-stranded DNA molecule with no apparent limits in nucleotide sequence or size.

As a model pathogen, EBOV was the primary choice. Firstly because a severe Ebola outbreak was taking place in African countries at the beginning of this project and at that time no approved prophylactic or therapeutic protocols were available. Secondly, EBOV it is classified as a Category A priority pathogen by NIAID and a Category A agent of bioterrorism by the CDC.

The EBOV genome is a single negative-strand RNA molecule encoding seven structural proteins, among which EBOV GP is a type I transmembrane glycoprotein of 676 amino acids in length and its transcript is made by an unusual transcriptional editing [23]. Full length GP has been shown to be responsible for eliciting a protective humoral immune response in infected individuals [8]. The cleavage of surface GP, by cellular metalloprotease, tumor necrosis factor α-converting enzyme (TACE), generates shed GP [21], which contributes to the host protective immune response [22]. Firstly, in silico customized full length GP ORF was successfully expressed in eukaryotic cells with a suitable expression vector and then integrated in BAC BoHV-4 genome through homologous recombination. BoHV-4-syEBOVgD106ΔTK replicated in cell culture at the same extent as the parental virus, thus no detrimental effect induced by the topological location of the foreign DNA in the BoHV-4 genome was observed. BoHV-4 is a *Gammaherpesvirus* belonging to the *Rhadinovirus* genera. Although BoHV-4 natural host is cattle, the virus has been isolated from other ruminants, including zebu (*Bos indicus*), American bison (*Bison bison*), African buffalo (*Syncerus caffer*), and sheep. Like other *Herpesviruses*, BoHV-4 is able to establish persistent infection in cells of the monocyte/macrophage lineage [24, 25] and in a bovine macrophage cell line (BoMAC) [26]. Furthermore, BoHV-4 experimental inoculation in rabbit demonstrates how spleen, as well as macrophages, are the main site of viral persistence [27]. Due to the lack of a direct correlation between BoHV-4 infection and specific lesions or pathology, BoHV-4 is not considered a primary pathogen and its genome was cloned as bacterial artificial chromosome (BAC), in order to be exploited as a gene delivery vector for immunization purposes and oncolysis. BoHV-4-based vector delivering antigens have been employed to immunize mice [9], rats [11], rabbits [12], sheep [13], swine [14], cows (paper in preparation) and goats [15] without any associated clinical signs or pathology.

Although goats are not susceptible to EBOV infection, BoHV-4-syEBOVgD106ΔTK immunization was performed in adults goats as they have previously been shown to be an appropriate large animal model for BoHV-4-based vector immunization [15]. Further, goats could be exploited as a source of antibodies production for antibody-based therapeutic in post-exposure treatment of EBOV disease. A pair of newly published studies [28, 29], demonstrated the efficacy of an ovine polyclonal antibody therapy against EBOV disease when tested in the stringent guinea pig model of EBOV disease.

The sub-cutaneous route of BoHV-4-based vector administration was shown to facilitate antigen production and vector replication takes place only at the site of inoculation [15], without spreading to the rest of the animal body. No BoHV-4-syEBOVgD106ΔTK viremia was detected in inoculated animals, although all BoHV-4-syEBOVgD106ΔTK inoculated animals were successfully immunized and high titers of EBOV GP antibodies were detected. The ability of BoHV-4-syEBOVgD106ΔTK to efficiently transduce goat skin cells, which consecutively expressed large amounts of GP, explains the consistent titer of antibodies produced and detected by the ELISA assay.

Whether the antibodies induced by BoHV-4-syEBOVgD106ΔTK in goats serum, correlates with a potential protection following a passive transfer in a suitable stringent model of EBOV disease remains unknown. Based on previous studies, which demonstrated the ability of BoHV-4 based vectors to efficiently protect against the Category A agent Monkeypox virus [10], it can be assumed that the use of BoHV-4 vector based platform represents an effective tool to test unknown antigens and vectors for class A pathogens.

## Conclusion

In this study, the possibility to generate a BoHV-4-based vector delivering an immune-dominant antigen coming from EBOV and proven to be able to generate high titer of antibodies in inoculated goats was shown.

## Additional files

**Additional file 1: Figure S1.** Overall strategy employed for cloning and expression of EBOV GP as a soluble secreted form. **A** Diagram (not to scale) showing the structure of gD106 tagged (red) EBOV GP. The EBOsecgD106 has been obtained by eliminating the transmembrane domain (TM). EBOsecgD106 peptide has been produced in serum free medium of HEK 293T cells transfected with pCMVEBOsecgD106. **B** A time course to optimize EBOsecgD106 expression at different time post transfection as analyzed by western immunoblotting was used. Twenty-four hours was considered as the best time post transfection to collect the cell sovranatant.

**Additional file 2: Figure S2.** Incorporation of EBOV GP into recombinant BoHV-4 particles. Extracts of purified viruses (BoHV-4-A and BoHV-4-syEBOVgD106) analyzed by Western immunoblotting.

## Abbreviations

EBOV: Ebola virus
BoHV-4: bovine herpesvirus 4
GP: glycoprotein
ELISA: enzyme linked immunosorbent assay
ORF: open reading frame
CFR: case fatality report
rVSV: recombinant vesicular stomatitis virus
ZEBOVΔG: Zaire EBOV glycoprotein
rAd5: recombinant adenovirus serotype 5
rChAd3: recombinant chimpanzee adenovirus serotype 3
rHPIV3: recombinant human parainfluenza virus 3
BAC: bacterial artificial chromosome
BSL: biosafety level
BEK: bovine embryo kidney
HEK 293T: human embryo kidney 293T
EMEM: Eagle’s minimal essential medium
FBS: fetal bovine serum
EBOsec: EBOV secreted fragment
PEI: polyethylenimine
DMEM: Dulbecco’s modified essential medium
MOI: multiplicity of infection
TCID_50_: tissue culture infectious doses 50
CPE: cytopathic effect
SDS-PAGE: sodium dodecyl sulfate-polyacrylamide gel electrophoresis
BoHV-1: bovine herpesvirus 1
HRP: horseradish peroxidase
LB: Luria–Bertani
BSA: bovine serum albumin
TMB: tetramethylbenzidine
TK: thymidine kinase
TACE: tumor necrosis factor α-converting enzyme
PI: pre-immune
BoMAC: bovine macrophage cell line
FDA: Food and Drug Administration
PA: polyadenylation
