# Supplementary material for: BoHV-4-based vector delivering Ebola virus surface glycoprotein
Source: J Transl Med. 2016 Nov 24;14:325. doi: 10.1186/s12967-016-1084-5 (PMC5122150; doi:10.1186/s12967-016-1084-5)
Supplement: Supplementary file 1 — Additional file 1: Figure S1. Overall strategy employed for cloning and expression of EBOV GP as a soluble secreted form. A Diagram (not to scale) showing the structure of gD106 tagged (red) EBOV GP. The EBOsecgD106 has been obtained by eliminating the transmembrane domain (TM). EBOsecgD106 peptide has been produced in serum free medium of HEK 293T cells transfected with pCMVEBOsecgD106. B A time course to optimize EBOsecgD106 expression at different time post transfection as analyzed by western immunoblotting was used. Twenty-four hours was considered as the best time post transfection to collect the cell sovranatant. [file 12967_2016_1084_MOESM1_ESM.pdf]

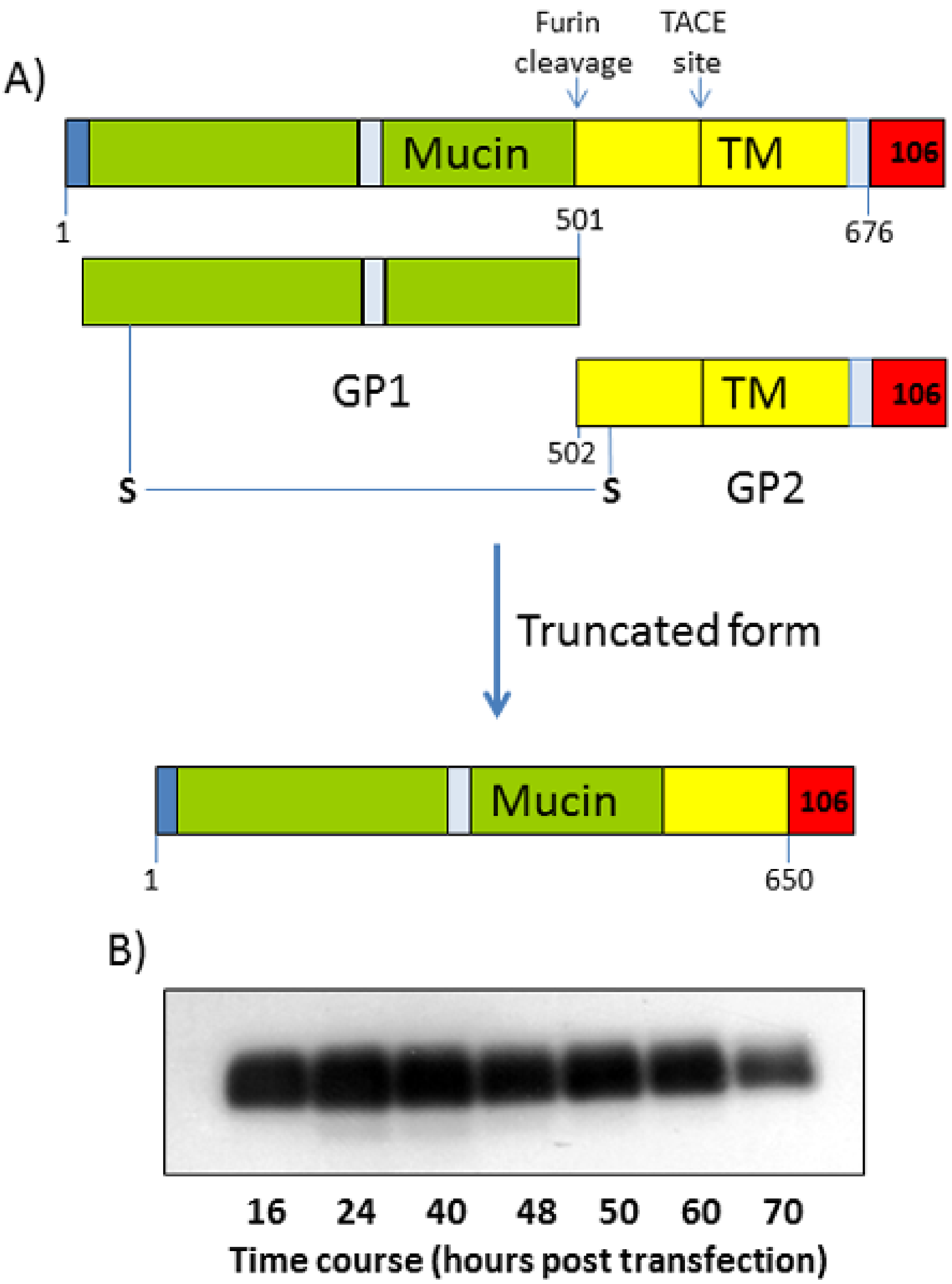

Supplementary Fig. 1. Overall strategy employed for cloning and expression of EBOV GP as a soluble secreted form. **A)** Diagram (not to scale) showing the structure of gD106 tagged (red) EBOV GP. The EBOsecgD106 has been obtained by eliminating the trasmembrane domain (TM). EBOsecgD106 peptide has been produced in serum free medium of HEK 293T cells transfected with pCMVEBOsecgD106. **B)** A time course to optimize EBOsecgD106 expression at different time post transfection as analyzed by western immunoblotting was used. Twenty-four hours was considered as the best time post transfection to collect the cell sovranatant.
