# Supplementary material for: BoHV-4-based vector delivering Ebola virus surface glycoprotein
Source: J Transl Med. 2016 Nov 24;14:325. doi: 10.1186/s12967-016-1084-5 (PMC5122150; doi:10.1186/s12967-016-1084-5)
Supplement: Supplementary file 2 — Additional file 2: Figure S2. Incorporation of EBOV GP into recombinant BoHV-4 particles. Extracts of purified viruses (BoHV-4-A and BoHV-4-syEBOVgD106) analyzed by Western immunoblotting. [file 12967_2016_1084_MOESM2_ESM.pdf]

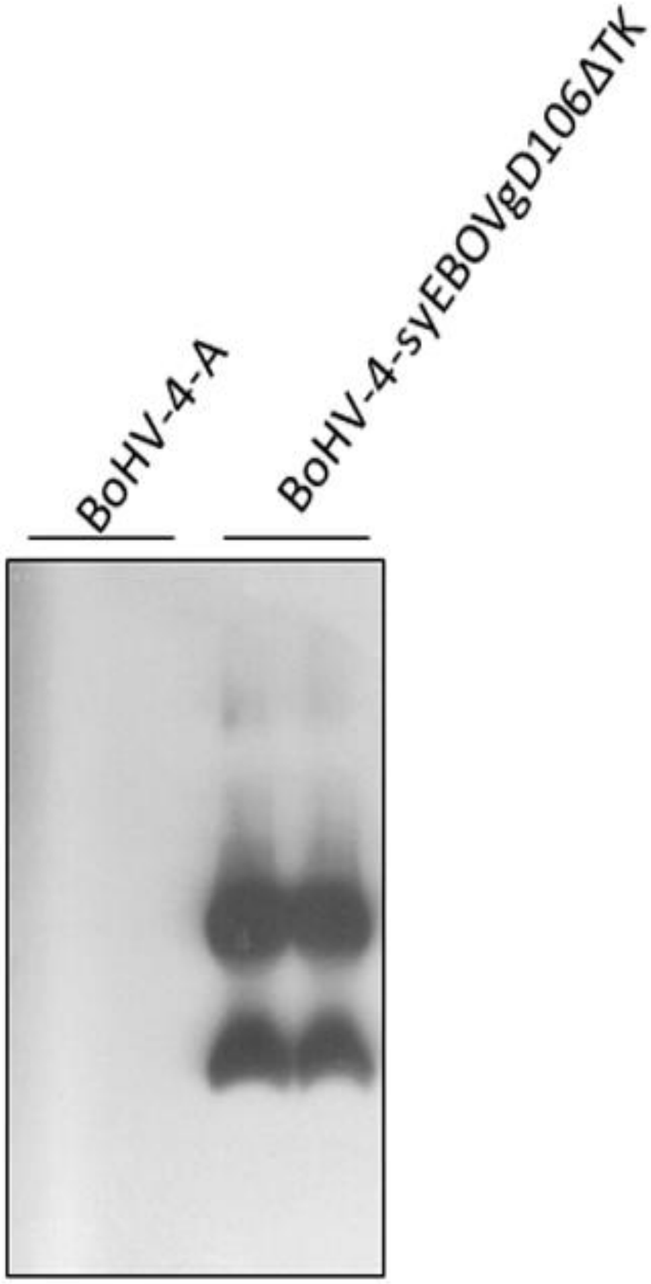

Supplementary Figure 2. Incorporation of EBOV GP into recombinant BoHV-4 particles. Extracts of purified viruses (BoHV-4-A and BoHV-4-syEBOVgD106) analyzed by Western immunoblotting.
